# Possible Mechanisms Underlying Aging-Related Changes in Early Diastolic Filling and Long Axis Motion—Left Ventricular Length and Blood Pressure

**DOI:** 10.1371/journal.pone.0158302

**Published:** 2016-06-28

**Authors:** Roger E. Peverill, Bon Chou, Lesley Donelan, Philip M. Mottram, John S. Gelman

**Affiliations:** Monash Cardiovascular Research Centre, Monash Heart and Department of Medicine (School of Clinical Sciences at Monash Medical Centre), Monash University and Monash Health, Clayton, Victoria, Australia; Harbin Medical University, CHINA

## Abstract

**Background:**

The transmitral E wave and the peak velocity of early diastolic mitral annular motion (e`) both decrease with age, but the mechanisms underlying these age-related changes are incompletely understood. This study investigated the possible contributions of blood pressure (BP) and left ventricular end-diastolic length (LVEDL) to age-related reductions in E and e`.

**Methods:**

The study group were 82 healthy adult subjects <55 years of age who were not obese or hypertensive. Transmitral flow and mitral annular motion were recorded using pulsed-wave Doppler. LVEDL was measured from the mitral annular plane to the apical endocardium.

**Results:**

Age was positively correlated with diastolic BP and septal wall thickness (SWT), inversely correlated with LVEDL (β = -0.25) after adjustment for sex and body surface area, but was not related to left ventricular end-diastolic diameter (LVEDD). Age was also inversely correlated with E (r = -0.36), septal e`(r = -0.53) and lateral e`(r = -0.53). On multivariable analysis, E was inversely correlated with diastolic BP and LVEDD, septal e`was inversely correlated with diastolic BP and positively correlated with SWT and LVEDL, after adjusting for body mass index, whilst lateral e`was inversely correlated with diastolic BP and positively correlated with LVEDL.

**Conclusion:**

The above findings are consistent with higher BP being a contributor to age-related reductions in both E and e`and shortening of LVEDL with age being a contributor to the age-related reduction in e`. An implication of these findings is that slowing of myocyte relaxation is unlikely to be the sole, and may not be the main, mechanism underlying age-related decreases in E and e`.

## Introduction

There is a progressive change in the pattern of left ventricular (LV) filling during normal aging which begins early in adult life [1,2], and results in substantial differences in LV filling between young and elderly subjects [3–6]. Studies using Doppler echocardiography have demonstrated an aging-related reduction in the velocity time integral (E_VTI_) [4,5] and peak velocity (E) [1,4–12] of the early diastolic transmitral flow signal, in conjunction with prolongation of the deceleration time (DT) [1,3–5,7,10,12,13] and the isovolumic relaxation time (IVRT) [1,5–7,11,14]. Aging is also associated with a gradual reduction in the peak velocity of LV long axis early diastolic motion (e`) [6,9–12,15–17], an echocardiographic variable now in common use for the assessment of LV relaxation, and as a correction factor for E in the estimation of mean left atrial (LA) pressure [18,19].

Although it has been more than 25 years since an effect of age on LV filling was first described [7], there has been ongoing uncertainty regarding the mechanisms underlying this effect [1,2,6,20–22]. While the LA–LV pressure difference provides the force which generates transmitral flow [23], and thus E and E_VTI,_ and age-related changes in E appear to be independent of LA pressure [2] there is limited and conflicting invasive data regarding the effects of aging on LV pressure fall [24,25]. Indeed, the largest available study reported that the time constant of relaxation (tau), a calculated variable in common use to describe the rapidity of LV pressure fall during isovolumic relaxation, does not increase with age [25]. While e`has been promoted as a non-invasive measure of LV relaxation [26], e`appears to be only a weak correlate of LV isovolumic relaxation (as determined by tau) in subjects with a normal ejection fraction [19], and whether this might reflect limitations of e`, tau, or possibly both, for describing the process of LV relaxation is unclear. In addition, consistent evidence that there is a much closer relationship of age with e`(r^2^ = 0.44–0.56) than E (r^2^ = 0.09–0.22) [1,12,27] suggests that there are likely to be differences in the mechanisms underlying aging-related reductions of long axis early diastolic motion and LV filling.

Blood pressure needs to be considered as a possible contributing factor to age-related decreases in early diastolic filling and motion as higher blood pressure (BP) is not only associated with a lower E [28–32] and e`[32–36], but BP increases with age [1,37], even in the absence of hypertension [2,38,39]. Age-related LV shape change also merits consideration as a factor as there is evidence that long axis LV function may be dependent on LV end-diastolic length (LVEDL) [37,40,41], and that LVEDL decreases during aging [37,42]. However, whether there could be independent contributions of BP and LVEDL to the age-related changes in early diastolic LV filling and long-axis motion is not known. Accordingly, in this study we have investigated the relationships of BP, LVEDL and age with E_VTI_, E, IVRT, DT and e`. On the basis that how far the annulus moves (i.e. the amplitude of its excursion) is not only likely to be closely related to the peak velocity of motion, but may be directly related to LV length, we also examined the relationships of BP, LVEDL and age with the amplitude of early diastolic excursion. To reduce the potential for heterogeneity and pathology within the study group, we only included adults of age <55 years who were free of cardiac disease and were not obese or hypertensive.

## Methods

### Subjects

All research involving human participants was approved by the Monash Health Human Research Ethics Committee and all clinical investigation was conducted according to the principles expressed in the Declaration of Helsinki. Written informed consent was obtained from all participants. The study group comprised 82 healthy adult subjects between the ages of 20 and 52 years. Subjects were not eligible if they had a history of diabetes, cardiac disease or hypertension. Height and weight were measured immediately prior to the echocardiographic study. Body surface area (BSA) was calculated using the formula: 0.0001 x 71.84 x (weight [kg])^0.425^ x (height [cm])^0.725^. Body mass index (BMI) was calculated as weight in kilogram per square metre in height (kg/m^2^) and subjects were excluded if they had a BMI > 30 kg/m^2^. The BP was measured at the end of the echocardiographic study with the patient resting in a supine position and subjects were excluded if they had a systolic BP >140 or a diastolic BP>90 mmHg. Mean BP was calculated as 2/3 x diastolic BP + 1/3 x systolic BP. All subjects had a normal LV ejection fraction (≥55%) and no more than mild valvular disease.

### Echocardiography

Echocardiography was performed by one of two technologists using a Sonos 5500 ultrasound machine (Philips, Amsterdam, The Netherlands). The studies were stored digitally and were measured offline using Xcelera V1.2 L4 SP2 (Philips, Amsterdam, The Netherlands) by one of two experienced investigators (BC or LD). LV M-mode recordings were obtained in the parasternal long axis view just distal to the mitral valve leaflet tips after alignment of the cursor perpendicular to the LV wall. 2-dimensional (2D) images were also used to facilitate identification of the endocardium and standard LV M-mode measurements were made [43], including LV end-diastolic diameter (LVEDD), septal wall thickness (SWT) and posterior wall thickness (PWT). Relative wall thickness (RWT) was calculated as 2 x PWT/LVEDD and LV mass was calculated using the modified formula of Devereux et al [44] and indexed to BSA. Apical 4- and 2-chamber 2-D loops of LV contraction were recorded, with attention paid to avoiding LV foreshortening, and used for off-line measurement of LV end-diastolic volume (LVEDV), end-systolic volume and the calculation of ejection fraction (LVEF) using the biplane method of discs. The length of the LV at end-diastole from the plane of the mitral annulus to the apical endocardium in the 4- and 2-chamber views was recorded during the measurement of the LVEDV, and the longest dimension from these 2 views has been used as the LVEDL [40]. LVEDL data was acquired as described above in all subjects by one investigator (RP) and this acquisition was repeated independently by a second investigator (LD). LVEDL measurements were made with investigators blinded to other echocardiographic measurements and subject data, and results obtained by the two investigators were compared.

LV inflow velocities were recorded using pulsed-wave (PW) Doppler in the apical 4-chamber view with the sample volume at the level of the mitral leaflet tips. For the early diastolic signal E_VTI_ was traced and E and DT were measured and for the late diastolic signal the peak velocity (A) was measured. Only one subject was not suitable for measurement of E_VTI_ because of partial fusion of the E and A waves. IVRT was measured as the interval between the mid-point of the aortic valve closure signal and the onset of transmitral flow using continuous wave Doppler oriented through the left ventricular outflow tract in the apical 5 chamber view.

**Fig 1:**
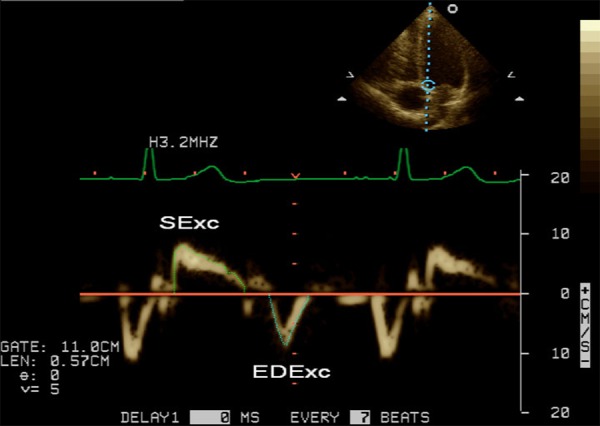
Example of the method for tracing the velocity time integrals of the systolic and early diastolic tissue Doppler signals from the mitral annulus.

PW tissue Doppler imaging (TDI) was performed in the apical 4-chamber view as previously described [45]. TDI velocities of longitudinal mitral annular motion were recorded during non-forced end-expiration apnoea at the septal and lateral annulus borders after optimizing parallel alignment of the ultrasound beam. Spectral PW Doppler was used with standard instrument settings and a sample volume length of 0.38–0.57 cm. Measurement of e' was performed as previously described [45]. Mitral annular excursion during systole (SExc), early diastole (EDExc) and atrial contraction (AExc) was assessed by measurement of the velocity time integrals of the respective signals by tracing just inside the outer border of the Doppler envelopes (Fig 1). An excellent correlation between mitral annular excursion by M-mode and the velocity time integral of the corresponding TDI signal has been previously reported [46]. The heart rate was calculated from the R-R intervals of the relevant TDI signals. All Doppler measurements are the average of 3 consecutive cardiac cycles.

### Statistical analysis

Statistical analysis was performed with Systat V13 (Systat Software, Chicago, IL). Continuous variables are presented as mean ± SD. Univariable regression analysis was performed to investigate the relationships of age with anthropometric measures and BP. Diastolic BP was used in most of the analyses as it was either similarly or more closely correlated to the dependent variables as mean or systolic BP. Univariable and multivariable linear regression analyses were performed to investigate the relationships of LVEDL with anthropometric measures, age and LVEDD. Univariable linear regression was performed to assess the relationships of transmitral flow variables and mitral annular TDI early diastolic velocities and excursion with BP, LVEDL and age and multivariable linear regression models were constructed to assess the independence of the observed relationships. BMI was also included in the multivariable analyses as adiposity tends to increase with age [47], increased adiposity appears to have adverse effects on the myocardium [48] and there is evidence of an inverse relationship of BMI with both E [49] and e`[45,50,51]. Given that age was expected to be correlated with the other variables of interest (BMI, BP and LVEDL), age was not added as an independent variable until the last step in the multivariable models. The partial correlation coefficient (β) value is provided for selected multivariable analyses. The adjusted value for r^2^ was used to estimate the degree of variability in a dependent variable explained by multivariable models. Apart from decisions regarding inclusion of variables in multivariable models, a p value of <0.05 was considered significant.

## Results

### Subject characteristics

The clinical characteristics and echocardiographic variables regarding LV structure and ejection fraction of the study subjects are shown in Table 1. Despite exclusion of subjects with obesity from this cohort there was a positive correlation of age with BMI (r = 0.29, p = 0.009), and despite exclusion of subjects with hypertension there were positive correlations of age with systolic (r = 0.22, p = 0.049) and diastolic BP (r = 0.46, p<0.001). There were also positive correlations of BMI with systolic (r = 0.41, p<0.001) and diastolic BP (r = 0.39, p<0.001), but no correlations of pulse pressure with either age or BMI.

**Table 1:** Clinical and left ventricular echocardiographic variables of the study group.

Sex (male: female)	37:45
Age (years)	32±9
Height (cm)	170±8
Weight (kg)	68±11
BSA (m^2^)	1.79±0.18
BMI (kg/m^2^)	23.3±2.5
Heart rate (bpm)	63±10
Systolic BP (mmHg)	112±12
Diastolic BP (mmHg)	68±9
Mean BP (mmHg)	83±9
Pulse pressure (mmHg)	45±10
LVEDD (cm)	5.0±0.5
SWT (cm)	0.8±0.1
RWT	0.31±0.04
LVMI (g/m^2^)	
*Males*	84±17
*Females*	66±15
LVEDL (cm)	
*Males*	9.6±0.7
*Females*	8.7±0.5
LVEF (%)	65±5

BSA = body surface area, BMI = body mass index, BP = blood pressure, LVEDD = left ventricular end-diastolic diameter, LVMI = left ventricular mass index, LVEF = left ventricular ejection fraction, LVEDL = left ventricular end-diastolic length, RWT = relative wall thickness, SWT = septal wall thickness

### Correlates of left ventricular structural measures

The univariable correlations of LVEDL with age, anthropometric measures and LVEDD are shown in Table 2. LVEDL was greater in males than females and was positively correlated with height, weight, BSA and BMI. While LVEDL was not correlated with age on univariable analysis, an inverse correlation of LVEDL with age became evident after adjusting for either sex or BSA (p<0.05 for either). There was no correlation of BP with LVEDL, with or without adjustment for sex and BSA (p>0.10 for all). On multivariable analysis including age, sex and BSA (Table 2), male sex was an independent predictor of a longer LVEDL and LVEDL was positively correlated with BSA and inversely correlated with age; together age, sex and BSA explained 50% of the variability in LVEDL. Age also remained a significant inverse correlate of LVEDL after adjustment for BSA during separate analysis of males and females (β = -0.31 & β = -0.32, respectively, p<0.05 for each). The LVEDL measurements from the two observers (9.0±0.7 v 9.1±0.7 cm) were similar and the partial correlation coefficients of age with LVEDL after adjusting for sex and BSA were similar and significant using the LVEDL data from either investigator (β = -0.24 v -0.25, p<0.005 for each).

**Table 2:** Univariable and multivariable correlates of left ventricular end-diastolic length.

	Univariable correlation coefficients		Multivariable correlation coefficients	
	r	P	β	P
Age	-0.16	0.16	-0.25	0.003
Male sex	0.59	<0.001	0.27	0.014
Height	0.57	<0.001		
Weight	0.62	<0.001		
BSA	0.64	<0.001	0.49	<0.001
BMI	0.43	<0.001		
LVEDD	0.55	<0.001		

BSA = body surface area, BMI = body mass index, BP = blood pressure, LVEDD = left ventricular end-diastolic diameter

LVEDV multivariable correlations were similar to those of LVEDL; there was an inverse correlation of LVEDV with age (β = -0.39, p<0.001) and a positive correlation with BSA (β = 0.55, p<0.001) after adjustment for sex, but no correlation with BP. LVEDD was also positively correlated with height, weight and BSA and was greater in males than females (p<0.01 for all), but while LVEDL was correlated with LVEDD, LVEDD was not correlated with age on univariable analysis, or after adjusting for sex, BSA or both (p >0.05 in all multivariable models).

### Standard Doppler and TDI variables–univariable and multivariable correlations

**Fig 2:**
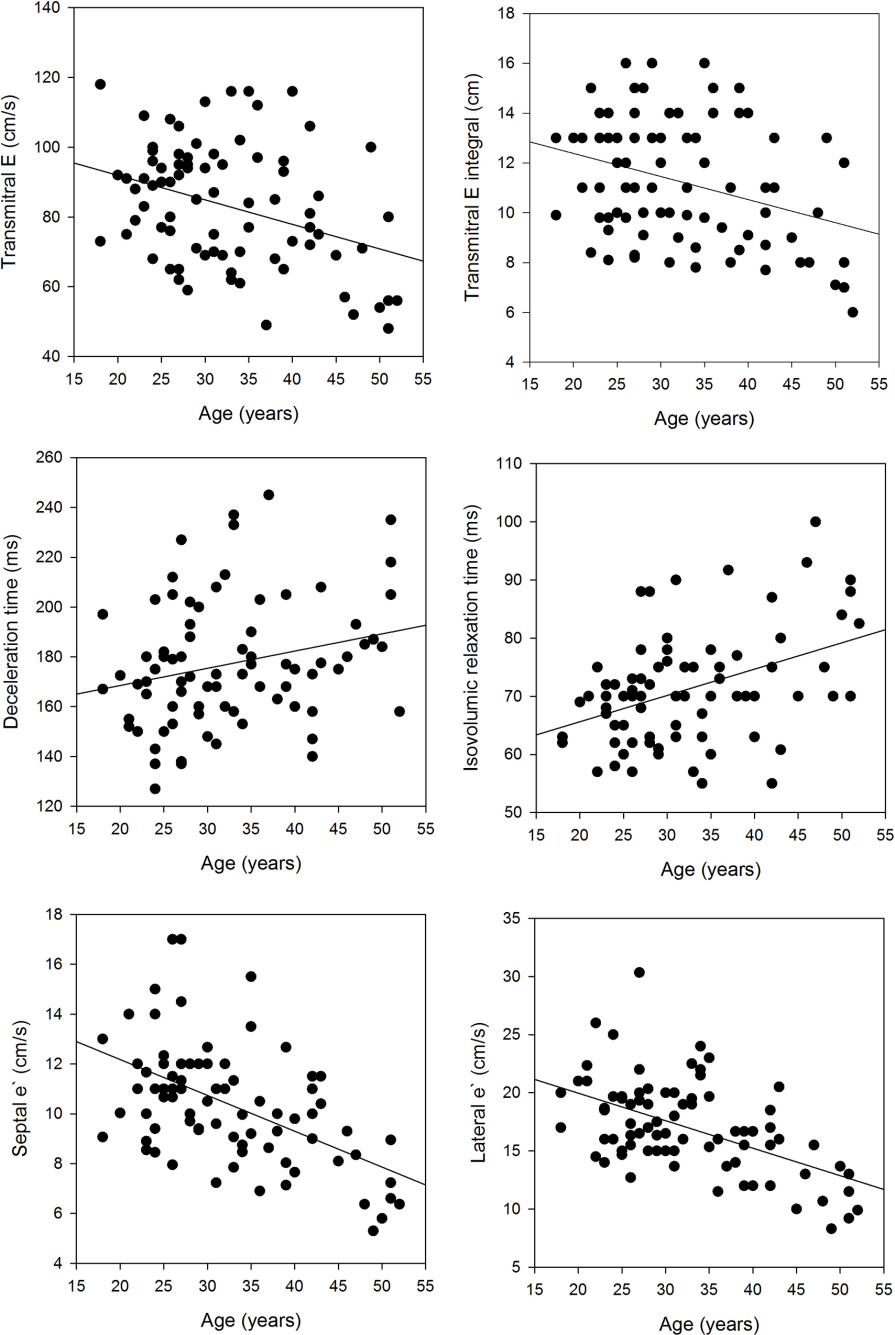
Scatter plots of selected echocardiographic variables and age. The linear regression statistics are shown in Table 3.

The mean values of the transmitral Doppler and TDI variables and the univariable correlations of these variables with age are shown in Table 3 and selected scatter plots are shown in Fig 2. Consistent with previous studies, age was inversely correlated with E_VTI_, E, E/A and e`and was positively correlated with DT, IVRT and A. Age accounted for the variability in the following Doppler variables: 28% of septal and lateral e`, 17% of septal EDExc, 20% of lateral EDExc, 18% of IVRT, 13% of E_,_ 11% of E_VTI_ and 6% of DT. The relationships of age with mitral annular excursion during systole and atrial contraction are also shown in Table 3. There were positive correlations of age with septal and lateral AExc but no correlations of age with septal or lateral SExc.

**Table 3:** Mean values and correlation with age of Doppler echocardiographic measurements.

	Mean ± SD	r	p value
E (cm/s)	83±17	-0.36	0.001
E_VTI_ (cm)	11.2±2.5	-0.33	0.002
A (cm/s)	48±12	0.29	0.008
E/A	1.89±0.58	-0.47	<0.001
IVRT (ms)	71.2±9.4	0.42	<0.001
DT (ms)	177±25	0.24	0.027
Septal EDExc (cm)	0.88±0.18	-0.41	<0.001
Septal e`(cm/s)	10.4±2.4	-0.53	<0.001
Lateral EDExc (cm)	1.22±0.23	-0.45	<0.001
Lateral e`(cm/s)	17.0±4.0	-0.53	<0.001
Septal SExc (cm)	1.38±0.20	-0.13	0.26
Lateral SExc (cm)	1.69±0.30	-0.11	0.33
Septal AExc	0.52±0.11	0.43	<0.001
Lateral AExc	0.50±0.13	0.50	<0.001

A = peak velocity of transmitral flow during atrial contraction, AExc = atrial contraction mediated excursion of the mitral annulus, DT = deceleration time, EDExc = early diastolic mitral annular excursion, e`= peak velocity of early diastolic mitral annular motion, E_VTI_ = velocity time integral of transmitral early diastolic flow signal, E = peak velocity of transmitral early diastolic flow, IVRT = isovolumic relaxation time, SExc = systolic excursion of the mitral annulus

#### E_VTI_ and E

The univariable correlations of E_VTI_ and E are shown in Table 4. E_VTI_ was inversely correlated with diastolic BP, but not related to sex, any of the anthropometric measures, or with LVEDD, SWT or RWT. Similar to E_VTI_, E was also inversely correlated with diastolic BP, but in contrast to E_VTI_, it was lower in males than females and inversely correlated with all the anthropometric measures, as well as with LVEDD, SWT and RWT. Neither E_VTI_ nor E were correlated with LEDVL. On multivariable analysis, there was an increase in the variability of E_VTI_ explained from 8 to 12% with the addition of age to diastolic BP, and diastolic BP was only borderline significant in this model (p = 0.09). On multivariable analysis, E was independently correlated with diastolic BP (β = -0.35, p = 0.001) and LVEDD (β = -0.26, p = 0.01), and this model explained 17% of the variability of E. Neither SWT nor RWT were significant contributors to models of E which included both diastolic BP and LVEDD. Addition of age to diastolic BP and LVEDD resulted in an increase in the variability of E explained to 21% and although there was a reduction in contribution of diastolic BP to the model, diastolic BP (β = -0.24, p = 0.035), LVEDD (β = -0.27, p = 0.008) and age (β = -0.26, p = 0.024) were all significant contributors. There were no independent contributions of sex, BSA or BMI to models of E which included diastolic BP, LVEDD and age. As expected, E was positively correlated with E_VTI_ (r = 0.80, p<0.001) and on multivariable analysis, E was independently correlated with E_VTI_ (β = 0.78, p<0.001) and LVEDD (β = -0.16, p = 0.02), with this model explaining 65% of the variability in E. There were no independent contributions of age, sex, diastolic BP or LVEDL to models of E which included E_VTI_ and LVEDD.

**Table 4:** Univariable correlates of transmitral E_VTI_ and E.

	E_VTI_		E	
	r	*P*	r	*P*
Male sex	-0.10	NS	-0.23	0.04
Heart rate	-0.01	NS	0.06	NS
Systolic BP	-0.28	0.01	-0.32	0.004
Diastolic BP	-0.31	0.005	-0.35	0.002
Mean BP	-0.33	0.002	-0.37	0.001
Height	0.03	NS	-0.20	0.07
Weight	-0.05	NS	-0.30	0.007
BSA	-0.03	NS	-0.28	0.01
BMI	-0.10	NS	-0.27	0.014
LVEDD	-0.12	NS	-0.26	0.02
SWT	-0.21	0.067	-0.32	0.004
RWT	-0.18	NS	-0.22	0.04
LVEDL	0.12	NS	-0.07	NS

BMI = body mass index, BP = blood pressure, BSA = body surface area, E_VTI_ = velocity time integral of transmitral early diastolic flow signal, E = peak velocity of early diastolic transmitral flow, LVEDD = left ventricular end-diastolic diameter, LVEDL = left ventricular end-diastolic length, NS = not significant, RWT = relative wall thickness, SWT = septal wall thickness

#### DT and IVRT

The univariable correlations of DT and IVRT are shown in Table 5. There were positive correlations of DT with height, weight and BSA, but DT was not correlated with BP, BMI, LVEDD, SWT, RWT or LVEDL. Neither age nor BSA remained significant when included together in a multivariable model of DT (p>0.05 for both). IVRT was inversely related to heart rate and positively correlated with diastolic BP, SWT and RWT, but was not related to systolic BP, any of the anthropometric measures, or with either LVEDD or LVEDL. On multivariable analysis IVRT was independently related to heart rate (β = -0.30, p = 0.007), SWT (β = 0.23, p = 0.04) and diastolic BP (β = 0.25, p = 0.03), with this model explaining 20% of the variability in IVRT. Addition of age to heart rate, SWT and diastolic BP resulted in an increase to 26% of the variability of IVRT explained, with heart rate (β = -0.26, p = 0.015) and age (β = 0.30, p = 0.009) significant contributors to the model, but diastolic BP (β = 0.11, p = 0.33) and SWT (β = 0.18, p = 0.09) no longer significant.

**Table 5:** Univariable correlates of deceleration time and isovolumic relaxation time.

	DT		IVRT	
	r	*P*	r	*P*
Male sex	0.10	NS	0.14	NS
Heart rate	-0.08	NS	-0.28	0.01
Systolic BP	0.14	NS	0.11	NS
Diastolic BP	0.12	NS	0.25	0.027
Mean BP	0.14	NS	0.22	0.048
Height	0.22	0.049	0.06	NS
Weight	0.23	0.035	0.12	NS
BSA	0.25	0.027	0.12	NS
BMI	0.18	NS	0.16	NS
LVEDD	0.12	NS	0.15	NS
SWT	0.11	NS	0.36	0.001
RWT	0.07	NS	0.33	0.003
LVEDL	0.05	NS	-0.02	NS

BMI = body mass index, BP = blood pressure, BSA = body surface area, DT = deceleration time, IVRT = isovolumic relaxation time, LVEDD = left ventricular end-diastolic diameter, LVEDL = left ventricular end-diastolic length, NS = not significant, RWT = relative wall thickness, SWT = septal wall thickness

#### EDExc

The univariable correlations of septal and lateral EDExc with age are shown in Table 3 and univariable correlations with other variables are shown in Table 6. Both septal and lateral EDExc were inversely correlated with diastolic BP, but not with systolic BP, and neither were correlated with LVEDD. BMI was an inverse correlate of septal EDExc, but there were no significant correlations of any of the anthropometric measures with lateral EDExc. Septal EDExc was inversely correlated with SWT and RWT but not with LVEDL, whereas lateral EDExc was positively correlated with LVEDL but not with SWT or RWT. Neither septal nor lateral EDExc were correlated with LVEDD.

**Table 6:** Univariable correlations of early diastolic mitral annular excursion at the septal and lateral borders of the mitral annulus.

	Septal EDExc		Lateral EDExc	
	r	*P*	r	*P*
Male sex	-0.11	NS	0.07	NS
Heart rate	-0.16	NS	-0.18	NS
Systolic BP	-0.11	NS	-0.18	NS
Diastolic BP	-0.35	0.001	-0.30	0.006
Mean BP	-0.28	0.011	-0.28	0.01
Height	0.00	NS	0.06	NS
Weight	-0.16	NS	0.00	NS
BSA	-0.12	NS	0.03	NS
BMI	-0.24	0.029	-0.03	NS
LVEDD	0.04	NS	0.13	NS
SWT	-0.22	0.043	-0.02	NS
RWT	-0.33	0.003	-0.04	NS
LVEDL	0.10	NS	0.34	0.002

BMI = body mass index, BP = blood pressure, BSA = body surface area, EDExc = early diastolic mitral annular excursion, LVEDD = left ventricular end-diastolic diameter, LVEDL = left ventricular end-diastolic length, NS = not significant, RWT = relative wall thickness, SWT = septal wall thickness

Despite lack of significance on univariable analysis, LVEDL became a significant positive correlate of septal EDExc (β = 0.25, p = 0.038) when included in a multivariable model with BMI, and BMI was a significant inverse correlate of septal EDExc in this model (β = -0.35, p = 0.004). Addition of diastolic BP and SWT to LVEDL and BMI in the model increased the variability of septal EDExc explained from 9 to 15%, but only diastolic BP (β = -0.24, p = 0.039) and LVEDL (β = 0.27, p = 0.027) were significant contributors to the model. The addition of age resulted in a further slight increase in the adjusted r^2^ (0.18) but none of the variables were then significant.

Lateral EDExc was independently correlated with LVEDL (β = 0.37, p = 0.001) and diastolic BP (β = -0.34, p = 0.001). Inclusion of age in the model with LVEDL and diastolic BP resulted in an increase in the adjusted r^2^ from 0.21 to 0.27 and in this model age (β = -0.31, p = 0.007) and LVEDL (β = 0.31, p = 0.003) were both significant contributors to lateral EDExc, but the contribution of LVEDL was less and the contribution of diastolic BP was no longer significant (β = -0.19, p = 0.096).

**e`.** The univariable correlates of septal and lateral e`with age are shown in Table 3 and univariable correlations of septal and lateral e`with other variables are shown in Table 7. Septal and lateral e`were both inversely correlated with diastolic BP. Septal e`was inversely related to weight, BSA and BMI but not with height, whereas lateral e`was not correlated with any of the anthropometric measures. Male sex was a predictor of a lower septal e`, but not of a lower lateral e`. LVEDL was positively correlated with lateral e`but not with septal e`, whereas SWT and RWT were both inversely correlated with septal e`but not with lateral e`. No relationship of LVEDD with either septal or lateral e`was evident.

**Table 7:** Univariable correlations of septal and lateral e`.

	Septal e`		Lateral e`	
	r	*P*	r	*P*
Male sex	-0.23	0.038	-0.03	NS
Heart rate	0.02	NS	-0.10	NS
Systolic BP	-0.22	0.05	-0.22	0.050
Diastolic BP	-0.44	<0.001	-0.35	0.001
Mean BP	-0.38	<0.001	-0.33	0.002
Height	-0.11	NS	0.04	NS
Weight	-0.30	0.007	-0.07	NS
BSA	-0.24	0.028	-0.03	NS
BMI	-0.34	0.002	-0.13	NS
LVEDD	-0.13	NS	0.12	NS
SWT	-0.40	<0.001	-0.09	NS
RWT	-0.38	<0.001	-0.15	NS
LVEDL	0.00	NS	0.30	0.006

BMI = body mass index, BP = blood pressure, BSA = body surface area, e`= peak velocity of early diastolic mitral annular motion, LVEDD = left ventricular end-diastolic diameter, LVEDL = left ventricular end-diastolic length, NS = not significant, RWT = relative wall thickness, SWT = septal wall thickness

On multivariable analysis including diastolic BP, BMI, SWT and LVEDL, septal e`was inversely correlated with diastolic BP (β = -0.29, p = 0.008), SWT (β = -0.34, p = 0.003) and LVEDL (β = 0.24, p = 0.03) but BMI was not significant (β = -0.16, p = 0.19). Adding age to the model of septal e`with diastolic BP, BMI, SWT and LVEDL resulted in an increase in the adjusted r^2^ from 0.28 to 0.35, but only age (β = -0.32, p = 0.005) and SWT (β = -0.25, p = 0.032) remained significant in this model. On multivariable analysis, lateral e`was independently correlated with LVEDL (β = 0.34, p = 0.001) and diastolic BP (β = -0.38, p<0.001), and together LVEDL and diastolic BP explained 22% of the variability in lateral e`. Adding age to the model of lateral e`with LVEDL and diastolic BP increased the variability of lateral e`explained to 33%; age was a significant contributor to this model (β = -0.40, p<0.001), the contribution of LVEDL was less but remained significant (β = 0.25, p = 0.01), whereas the contribution of diastolic BP became only borderline significant (β = -0.19, p = 0.08). Thus, there was an additional independent effect of age on lateral e`which accounted for about 1/3 of the total variability of lateral e`not explained by BP and LVEDL in the model.

There were correlations between septal e`and septal EDExc (r = 0.80, p<0.001) and lateral e`and lateral EDExc (r = 0.73, p<0.001). After including septal and lateral EDExc in the respective models of septal and lateral e`, neither diastolic BP nor LVEDL remained significant correlates of e`. However, age remained an independent predictor of septal and lateral e`when combined in models with septal and lateral EDExc, respectively (p<0.01 for each). The variability of e`explained with the addition of age to EDExc increased from 64% to 69% for the septal wall and from 52% to 57% for the lateral wall.

## Discussion

The aim of this study in healthy young to middle aged adult subjects was to investigate possible contributions of BP and LVEDL to age-related changes in Doppler measures of early diastolic LV filling and long axis motion. BMI was considered in the analysis due to previous evidence of an inverse relationship of BMI with both E [49] and e`[45,50,51], the latter relationship described even in the absence of obesity [45]. LVEDD, SWT and RWT were also considered in the analysis due to previous evidence showing aging effects on short-axis remodeling [38,42,52,53]. We found inverse correlations of age with E, E_VTI_ and e`and positive correlations of age with DT and IVRT, consistent with previous studies which have reported that aging-related changes in these diastolic variables begin early in adult life [1,2]. Furthermore, that age explained 28% of the variability in septal and lateral e`but only 13% of the variability in E is consistent with previous findings indicating a disparity between the degree of correlation of age with e`and E [1,12,27], at the same time implying a difference in the mechanisms underlying aging effects on early diastolic filling and long axis motion. We also found positive correlations of age with BP and BMI (despite the exclusion of subjects with hypertension and obesity) and an inverse relationship between age and LVEDL, supporting the possibility that BP, LVEDL and BMI could account for at least a portion of the effects of aging on Doppler-derived diastolic variables in healthy individuals. There was no significant relationship of age with LVEDD, but consistent with previous evidence, SWT and RWT increased with age [52], indicating that LV short-axis remodeling also needed to be considered as a possible contributor to the age-related decreases in E and e`in this cohort.

The inverse relationship between age and LVEDL seen in our study was independent of other predictors of LVEDL, including sex, BSA and LVEDD. That the relationship between age and LVEDL was not seen on univariable analysis could relate to the modest degree of correlation between age and LVEDL, in conjunction with the limited cohort size, and possible interactions between LVEDL, BSA, age and sex. Nevertheless, our finding is consistent with previous cross-sectional data that LVEDL decreases, LVEDD is relatively stable and thus LV shape becomes more spherical during aging [37,42]. It is also an important addition to previous findings as it suggests that the effects of aging on LVEDL begin early in adult life. That LVEDV also decreased with age in our study group was expected given the reduction in LVEDL, even in the absence of any change in LVEDD, and is consistent with echocardiographic and cardiac magnetic resonance LV volume data in other cohorts of subjects without cardiac disease [38,42,53]. The ultrastructural basis of the reduction in LVEDL and LVEDV with age is not known, but there may be components of cardiomyocyte loss, increase in the volume of residual cardiomyocytes and increase in diffuse interstitial fibrosis. While the age-related change in LVEDL is seen in both men and women [42], there appear to be sex-related differences in the age-related increase in myocardial extracellular volume [54], as loss of myocytes during aging has been observed in men but not in women [55]. Whatever the ultrastructural basis, our finding provides support for the possibility that reduction in LVEDL with age could be a contributor to the aging-related changes in early diastolic LV long axis motion.

A lower E has been reported to occur in hypertension independently of LV structural change [56], an inverse correlation between E and BP, particularly with diastolic BP, has been reported in observational studies [8,28,30], and an acute decrease in E can be seen during acute elevation of BP in humans by angiotensin infusion [32]. In the present study E_VTI_ and E were both inversely correlated with diastolic BP, and to a lesser extent, with systolic BP. BP only accounted for a portion of the effect of age on E_VTI_ and E as the addition of age to diastolic BP in the multivariable models of E_VTI_ and E resulted in an improvement in the variability of E_VTI_ and E explained. No relationship of BP with E_VTI_ independent of age was demonstrated, as diastolic BP was no longer significant after inclusion of age in the model, but there was still a residual, albeit less close, correlation of diastolic BP with E following the addition of age. Although there were increases in SWT and RWT with age, there was no evidence for a contribution of LV short-axis remodeling to age effects on E_VTI_, and the correlation of diastolic BP with E remained significant after adjusting for either SWT or RWT_._ While the findings of the present study cannot prove that BP represents a casual mechanism rather than just an association of a lower E and E_VTI_, it is important that a similar inverse relationship of diastolic BP with E and E_VTI_ has been previously reported in normotensive adolescents, suggesting independence of BP effects from age [28].

There are at least two mechanisms which have been proposed for an acute effect of BP on early diastolic LV flow: (1) an afterload-mediated reduction of contraction and (2) an afterload-mediated delay or slowing of relaxation. An effect of BP on both contraction and E was demonstrated in a study in closed chest anaesthetized dogs which showed that both the early diastolic LA-LV gradient and E were related to end-systolic LV volume, and that end-systolic volume was not only determined by contractility but was also independently and positively correlated with diastolic BP [31]. As the rapidity of the fall in LV pressure in the normal heart is facilitated by elastic recoil and elastic recoil decreases in conjunction with increases in end-systolic volume, a higher BP can explain a reduced LA-LV gradient because increased afterload leads to a larger LV end-systolic volume [31,57]. On the other hand, alterations in LV relaxation have been demonstrated in animal studies during interventions which increase afterload during LV contraction [58,59]. Whether this effect is independent of changes in contraction is less clear as an increase in load beginning early in ejection delays the onset of LV pressure fall by prolonging contraction without changing the slope of LV pressure fall, whereas an increase in load beginning in the second half of ejection results in both a shortening of contraction time and a reduced slope of the LV pressure fall [59]. Similar findings have been reported in some [60,61] but not all [62] studies where afterload was modified in humans.

While not all observational studies have found a correlation of BP with E [1,35], the presence of such a relationship could be dependent on the subject cohort and also on the method of analysis. For example, the wall stress (afterload) of a left ventricle in subjects with hypertension and either concentric remodeling or concentric hypertrophy would not be as high as in a non-remodeled ventricle, and this could moderate the tendency to an increase of end-systolic volume with a higher BP. There are also statistical issues, with the inclusion of both age and BP in multivariable models of E potentially masking an effect of BP due to colinearity between age and BP. Moreover, the inclusion of elderly subjects in previous studies could confound the assessment of a potential relationship of diastolic BP with E as while both systolic and diastolic BP increase with age up until the age range of 50–60 years, diastolic BP starts to decrease as age progresses past 60 years [63]. All of these potential confounding factors were minimized in the present study as hypertension and thus hypertensive heart disease were exclusion criteria, only subjects <55 years were eligible for inclusion and age was not included till last in all multivariable analyses.

There were differences in the determinants of E_VTI_ and E, with only E higher in females and only E inversely and independently correlated with LVEDD. A higher E in females than males is consistent with previous studies [4,5,8], but the cause has not been certain. It is thus important that an effect of sex on E in the present study was no longer evident after inclusion of LVEDD in the model, and so a feasible explanation for the effect of sex on E may be the smaller LV short axis diameter (and accompanying smaller mitral annular) size in women [5]. That there could be differences in the determinants of E_VTI_ and E has received little attention in previous studies, but would not be surprising as E_VTI_ is likely to be more closely related to the volume of early diastolic filling than E. A relationship of BP with E was no longer evident after adjustment for E_VTI_, consistent with the underlying mechanism for the inverse correlation of BP with E being that a higher BP was associated with a reduction in the volume of early diastolic flow. Despite the inverse correlation of E with LVEDD, there was no relationship of LVEDL with either E_VTI_ or E, suggesting that age-related decreases in early diastolic flow are not due to LV long-axis remodeling.

There was a positive correlation between age and IVRT in our cohort and this finding is consistent with previous studies [1,5,7,64], however, the cause of the prolongation of IVRT with age has not been clear [1]. By first principles, IVRT will be dependent on the timing and magnitude of end-systolic BP (i.e. the aortic pressure at the time of aortic valve closure), the rapidity of the LV pressure drop following aortic valve closure and the crossover point of the LA and LV pressure curves, the last of these factors dependent on LA as well as LV pressure [65]. However, even with the current understanding that LA pressure does not appear to change with healthy aging [2,21], determining the cause of the prolongation of IVRT with age remains complicated as not only can the rate of LV pressure fall be affected by afterload, but as previously discussed, the relationship between BP and LV pressure fall is complex. In addition, the end-systolic BP is likely to be related to both systolic and diastolic BP, and thus would be expected to change with age.

We found a positive correlation of IVRT with diastolic BP (although not with systolic BP). A positive relation of IVRT with hypertension severity was recently reported in a large community study [66], and has also been reported in other [64,67] but not all previous observational studies [1,5]. That the effect of BP was only borderline significant when included with age is therefore consistent with diastolic BP explaining at least a portion of the effect of age on IVRT. We found an inverse correlation of IVRT with heart rate which was independent of both age and BP, a finding which is consistent with some [1,5] but not all previous studies [67]. Nevertheless, such a relationship was not unexpected given that acceleration of LV pressure fall has been described in association with pacing mediated increases in heart rate [68,69]. That there was no evidence of an increase in either E_VTI_ or E with higher heart rate suggests that LV pressure fall can accelerate without resulting in an increase in the early diastolic LA-LV gradient. Independent of heart rate and diastolic BP, SWT was also a positive correlate of IVRT and as age was positively correlated with SWT, short-axis remodeling is also a possible contributor to the age-related prolongation of IVRT. On the other hand, IVRT was not related to LVEDL in univariable or multivariable analyses, therefore not suggesting any effect of long axis LV remodeling on IVRT.

Diastolic BP was an inverse correlate of septal and lateral e`in the present study, and this is consistent with increasing evidence from both observational and interventional studies, and in both healthy and hypertensive subjects, of an inverse relationship between BP and e`[32–36]. That afterload might be a determinant of e`has also been suggested by the findings from two observational cross-sectional studies in humans, both of which reported an inverse relationship of e`with measures of late systolic load [70,71]. As e`must be related to, and will also be limited by, how far the mitral annulus moves during early diastole, in the present study we further investigated the relationship between BP and early diastolic motion by also examining the relationship with EDExc. Both septal and lateral EDExc were inversely correlated with diastolic BP and after adjusting for EDExc there was no independent relationship of BP with e`, suggesting that the mechanism underlying the BP relationship with e`is a decrease in early diastolic excursion, i.e. a higher BP is associated with a lower EDExc and therefore a lower e`. Similar to the relationship of E with BP, there are at least two possible mechanisms for the inverse relationship between e`and BP. One possible mechanism is that higher afterload leads to reduced long axis contraction and thus reduced early diastolic recoil [31]. Indeed, both s`[70,71] and systolic strain [71] have also been reported to vary inversely with late systolic load. While there was no relationship between BP and either s`or SExc at either annular border in the present study (results not shown), the absence of these relationships does not exclude a contribution of higher BP to reduced long axis contraction. Thus, long axis systolic excursion could be maintained despite an afterload mediated reduction in contraction by an increase in preload and utilization of the Frank-Starling mechanism, with preload best thought of in this circumstance as wall stretch due to atrial contraction [72]. Indeed, an age-related increase in LV long-axis stretch prior to contraction is evident in the positive correlation of age with AExc seen in the present study. On the other hand, a direct effect of afterload on LV relaxation is also possible and it is feasible that e`could be influenced by BP effects on both contraction and relaxation.

A linear relationship between LVEDL and e`has been reported previously based on colour flow TDI velocities obtained from healthy subjects and using averaged results from all LV walls [41]. By using pulsed-wave TDI and not averaging the results of the different walls, we were not only able to provide support for these previous findings, but we were able to investigate for possible differences in the behaviour of the septal and lateral LV walls, and also investigate for mechanisms underlying these relationships. Thus, while LVEDL was a positive correlate of lateral e`in the present study, no relationship with septal e`was evident on univariable analysis. Furthermore, we found LVEDL to be positively correlated with both lateral e`and EDExc and the relationship of LVEDL with lateral EDExc fully accounted for the relationship of LVEDL with lateral e`, as there was no independent relationship of LVEDL with lateral e`after including EDExc in the model of lateral e`. A potential explanation for the lack of univariable relationship of LVEDL with septal EDExc and septal e`was identified, with BMI a positive correlate of LVEDL but an inverse correlate of both septal EDExc and septal e`. An inverse relationship between BMI and septal e`has been previously described [50] and such a relationship has the potential to mask a positive relationship of septal e`with LVEDL. Indeed, in our cohort, LVEDL became a significant positive correlate of both septal EDExc and septal e`when included in models with BMI. There was also evidence of an effect of short-axis remodeling on septal long axis function as SWT was an inverse correlate of septal e`independent of LVEDL.

There have been a number of invasive studies which have investigated the mechanisms underlying the decreases in E and e`with aging. Two recent studies using right heart catheterization have provided strong evidence that the decreases in E and e`, and the prolongation of IVRT, seen with healthy aging are not due to a reduction of LA pressure [2,21]. A higher LV pressure during early diastole must therefore be the main cause of a lower early diastolic transmitral gradient and therefore the lower E_VTI_ and E which occurs with aging, and it has been assumed by some investigators that this higher LV pressure during early diastole is due to slowing of the LV pressure fall during isovolumic relaxation [26,73,74]. However, slowing of LV pressure fall with age was not confirmed in a study of a symptomatic group of 55 subjects between 20 and 77 years of age who were free of cardiac disease, and in which there was direct measurement of LV pressure using high fidelity catheters and calculation of tau [25]. As tau only reflects a portion of the process of LV relaxation and is also recognized to have other theoretical limitations [75–77], one possible explanation for this negative finding is that an age-related increase in LV pressure during early diastole may occur in the absence of prolongation of tau. While the ideal investigation to resolve these issues would be measurement of simultaneous LV and LA pressures using high fidelity catheters in healthy asymptomatic adult subjects of varying ages, the likelihood of such an investigation being performed is small given the ethical issues involved in the conduct of such a study. Therefore non-invasive techniques to investigate these questions, such as those used in the present study, remain of considerable importance.

Although e`is considered to be a non-invasive measure of LV relaxation, and an inverse correlation of e`with invasively measured tau has been reported in a number of human studies [19,78–80], the correlation appears to be weak (r^2^ = 0.08) in subjects with a normal ejection fraction [19], this group being of particular relevance to the effects of healthy aging on LV relaxation. Furthermore, as early diastolic long axis motion does not begin until after mitral valve opening, it has been pointed out previously that e`can only provide indirect information about LV isovolumic relaxation [81]. Thus, the finding in the present study, and in multiple previous studies, of an inverse correlation of age with e`cannot be considered to be conclusive evidence for a relationship of age with either tau or slowing of LV relaxation. As already mentioned, IVRT prolongation also cannot provide definitive evidence of slowing of isovolumic LV relaxation with age given that the onset of the isovolumic relaxation period is dependent on end-systolic pressure, which is likely to be affected by age, and which cannot be precisely determined using non-invasive techniques.

Slowing of myocyte relaxation has been proposed as the main mechanism for the age-related impairment of LV relaxation in humans [2,22], however, the findings of the present study are consistent with the possibility of contributions to age-related changes in E and e`by alternative mechanisms. Thus, the inverse correlations of both E and e`with BP are consistent with an afterload-related mechanism, which could act in part via a reduction in contraction [31]. Furthermore, the reduction of e`(and EDExc) can be partly accounted for by the concomitant reduction of LV length with age, a mechanism likely related to structural remodeling which also does not require that there be slowing of either LV or myocyte relaxation. While the above findings certainly do not exclude a contribution from a slowing of relaxation in cardiac myocytes, it is also important there is currently no direct evidence for an age-related slowing of relaxation in human cardiac myocytes [82], particularly for any age-related slowing beginning relatively early in adult life. Furthermore, while a reduction in the maximum velocity of relaxation has been shown in cardiac preparations from senescent animals [83,84], this is not an isolated diastolic abnormality, as both myocyte and papillary muscle studies show that older age is also accompanied by reductions of contraction amplitude and maximum contraction velocity [83–85]. There is also at least one other previously reported and likely myocyte-independent contributor to the age-related reduction in E, this being non-uniformity of regional LV short-axis diastolic motion [86].

There are a number of limitations of our study. As it is a cross-sectional study, there are limits to the conclusions which can be drawn regarding causality. Nevertheless, the findings of relationships of LVEDL with age and LV long axis function were not unexpected based on previous studies [37,40,42]. A further limitation of this study is that the study group was limited to healthy adult subjects <55 years and therefore the findings may not be applicable to older subjects or subjects with cardiac pathology. However, as previously discussed, the absence of elderly subjects was also an important component of the study design given the potential for confounding by the opposite changes in systolic and diastolic BP which start to occur during the age range of 50–60 years. The LVEDL reflects the length of the left ventricle from the mid-point of the mitral annulus to the apex and will be related to, but not the same as, the length of the relevant LV walls. Furthermore, LVEDL was measured by 2D echocardiography and this technique is unlikely to have been as accurate as cardiac magnetic resonance [42]. However, the use of a less accurate technique would in general be more likely to result in false negative than false positive findings. Finally, this was a relatively small study, and it is recommended that these findings be confirmed in other studies in healthy subjects.

## Conclusions

In this cross-sectional study of young to middle aged adult subjects, there was an age-related shortening of LVEDL and increase in SWT and RWT but no age effect on LVEDD. At least a portion of the age-related decreases in E_VTI_ and E and prolongation of IVRT in this cohort could be accounted for by an elevation of BP with age, despite hypertension being one of the exclusion criteria. The relation between E and E_VTI_ was partly explained by variation in LVEDD but there was no relationship of LVEDL with any of the standard Doppler measures related to transmitral flow. Major portions of the inverse relationships of age with e`and EDExc could be explained by a combination of the increase in BP and the reduction in LVEDL during aging. A direct structural relationship between LVEDL and early diastolic long axis motion (additive to the effect of BP) therefore provides a possible explanation for the greater correlation of age with e`than with E. A further implication of these findings is that a functional abnormality of active LV myocyte relaxation is unlikely to be the sole, and may not even be the main, mechanism for age-related reductions in early diastolic long axis motion. For e`in particular, possible mechanisms include LV long axis remodeling and afterload elevation, both resulting in a diminished extent of early diastolic excursion, and potentially explained in part by a decreased extent of contraction. Nevertheless, additional mechanisms for the reduction in e`with aging, which may include slowing of LV myocyte relaxation, are required given the additional contribution of age to models of EDExc and e`which included LVEDL and diastolic BP. The above findings have important implications for the clinical interpretation of E and e`in both the presence and absence of cardiac disease.

## Supporting Information

S1 File Excel file containing raw data. (XLSX)

## Abbreviations

A: peak velocity of transmitral flow during atrial contraction
AExc: atrial contraction mediated excursion of the mitral annulus
BMI: body mass index
BP: blood pressure
BSA: body surface area
DT: deceleration time
E: peak velocity of early diastolic transmitral flow
e`: peak velocity of early diastolic mitral annular motion
E_VTI_: velocity time integral of transmitral early diastolic flow signal
EDExc: early diastolic mitral annular excursion
IVRT: isovolumic relaxation time
LA: left atrial
LV: left ventricular
LVEDD: left ventricular end-diastolic diameter
LVEDL: left ventricular end-diastolic length
LVMI: left ventricular mass index
RWT: relative wall thickness
SExc: systolic mitral annular excursion
SWT: septal wall thickness
